# Soil-Transmitted Helminths in Top Soils Used for Horticultural Purposes in Cape Coast, Ghana

**DOI:** 10.1155/2018/5847439

**Published:** 2018-02-12

**Authors:** David Oscar Yawson, Isaac Benjamin Yao Kudu, Michael Osei Adu

**Affiliations:** ^1^Department of Environmental Science, University of Cape Coast, Cape Coast, Ghana; ^2^Nuclear Chemistry and Environmental Research Centre, Ghana Atomic Energy Commission, P.O. Box LG 80, Legon, Accra, Ghana; ^3^Department of Crop Science, University of Cape Coast, Cape Coast, Ghana

## Abstract

This paper investigated the concentrations of eggs of three helminths (roundworm, hookworm, and whipworm) in the so-called black soils used for domestic and urban landscaping, home gardening and as growth medium for potted plants and pot experiments. The black soils are largely collected from active or abandoned waste dumpsites and fallowed or vegetated idle sites in the urban fringe or rural areas. Users buy black soils from dealers. Samples of black soils used for various purposes and at different places were collected for analysis of helminth eggs. The Modified EPA Method, which combines flotation and sedimentation, was used to isolate the eggs. The results show that these black soils have substantial loads of helminth eggs, with roundworm being dominant, followed by hookworm. Mean concentrations of helminth eggs were 2.45 (roundworm), 1.38 (hookworm), and 0.25 (whipworm) g^−1^ soil, respectively. The helminth egg loads also declined with duration of use of the black soils. It is concluded that black soils used for horticultural purposes in Ghana can be a potential source of helminth infestation. Therefore, treatment of black soils, regulation of black soil market and use, and development of growth media industry should be important components of helminth control strategy.

## 1. Introduction

Soil-transmitted helminths (STH), also known as geohelminths [1, 2], are* “intestinal worms infecting humans that are transmitted through contaminated soil.”* The main soil-transmitted helminthiases include infections with roundworm* (Ascaris lumbricoides)*, hookworm (*Ancylostoma duodenale *and* Necator americanus*), and whipworm* (Trichuris trichiura)* [3]. The STH eggs normally get to the environment through the faeces of infected persons or animals and are normally taken up through skin contact (hookworm) and oral ingestion (roundworm and whipworm) [4–6]. Poor water and sanitation practices or conditions are a major risk factor for the distribution, infection, and prevalence of STH [3, 5, 7, 8]. Globally, over 1.5 billion people are infected with STH [4, 5, 7]. Collectively, the STH infection is the largest contributor to the disease burden of neglected tropical diseases [9, 10] and rivals that of the main high-mortality conditions such as HIV/AIDS and malaria [8]. It accounts for 85% of the neglected tropical disease burden for the poorest 500 million people in Sub-Saharan Africa [8]. Children have the highest prevalence and intensities of infections and are particularly vulnerable to STH infections which reduce physical and cognitive development [8, 11] and contribute to anemia [1, 8].

As the name implies, soils play a major role in the distribution and prevalence of STH. Soil is one of the main reservoirs of helminth eggs. Particularly, in areas with poor environmental sanitation, soils can be contaminated with large number of helminth eggs, which subsequently infect people through oral ingestion or direct skin contact [5, 6, 12, 13]. Studies on STH have largely focused on the prevalence and intensity of STH infections [12, 13], especially among children [11]. There is little information on STH loads of soils used for domestic landscaping or gardens in jurisdictions with weak growth media market or industry. In Ghana, nutrient-rich top soils (popularly known as “black soil”) are predominantly used for domestic and urban landscaping and demand is known to exceed supply in the big cities [14]. Recently, black soils are being used in home gardens, urban farms, and pot experiments. The black soils are normally supplied by dealers who collect these soils from active or abandoned refuse dumpsites [14] and fallowed areas or pristine top soils. Recently, top soils scrapped off from construction sites are also collected and sold as black soil, as well as top soil from any vegetated site that seems to lie idle. The market for black soil seems lucrative as the plant-growth media industry, if at all, is very young and weak. Given Ghana's challenges with access to improved sanitation [10, 15], with open defecation and free range animal husbandry systems still in practice in both urban and rural settings [10], the black soil can be a potential source of STH infection but this has not been studied in Ghana. This paper therefore determined the egg loads of the three main STH in black soil samples collected from various locations in Cape Coast in the Central Region of Ghana.

## 2. Methods

### 2.1. Soil Sampling

Soil samples were collected from various locations in Cape Coast where black soils have been used for various horticultural or research purposes. Sample sources included heaps of black soil at the School of Agriculture Teaching and Research Farm (University of Cape Coast), purposefully landscaped surfaces at homes, hostels, schools (primary and preschools), and few backyard gardens. The landscaped surfaces were either grassed or nongrassed, with or without other aesthetic plants, in the selected homes, hostels, and schools. These locations were selected purposively and opportunistically based on the presence of landscaped surfaces and willingness of property owners to allow soil sampling on their premises. At each location or source, two or three samples were randomly collected, bulked, and fractioned to obtain a representative sample. A total of 40 representative samples were collected and used for this study. Samples were collected in the morning hours, between six and nine o'clock. Samples were kept cool after collecting, transported to the laboratory, and analyzed for eggs of helminths.

### 2.2. Identification of Eggs of Helminths

The Modified EPA Method, which is a combination of flotation and sedimentation, as applied by [16], was used for the current study. For each soil sample, 30 g was weighed, pulsified, and washed in 2 L distilled water and subsequently sieved into 4 L container. Samples of the suspension were transferred into 2 L containers and allowed to settle overnight. This allows the eggs of the helminths to settle. The next day, the supernatant was sucked out as much as possible, leaving the sediments at the bottom of the containers. The sediments were then transferred into six 50 ml centrifuge tubes. The containers were rinsed with sterile water and the rinsed water was added to the centrifuge tubes to ensure complete transfer of the eggs. The sediments in the tubes were then centrifuged at 1500 rpm for three minutes.

Thereafter, the supernatant was gently poured away and about 150 ml ZnSO_4_ solution (specific gravity = 1.3) was added to sediments remaining at the bottom of the centrifuge tubes. This causes the eggs to float on the suspension. A sterile spatula was used to stir and homogenize the mixture which was then subject to centrifugation again at 1500 rpm for three minutes. The supernatant which contains the eggs was poured into the 2 L container and diluted with about 1 L of distilled water and allowed to settle for about three hours to get the eggs to resettle. The process of centrifugation was repeated by sucking off the supernatant and transferring the sediments into 50 ml centrifuge tubes, together with rinses of the 2 L container. The tubes were then centrifuged at 1500 rpm for three minutes. The sediments for each sample were transferred into a centrifuge tube and centrifuged at 1500 rpm for three minutes. A 15 ml acid/alcohol buffer solution (made from 5.16 ml of 0.1 N H_2_SO_4_ in 350 ml ethanol) and about 5 ml ethyl acetate were added to the sediment and shaken, occasionally letting out the gas. The mixture was then centrifuged at 2200 rpm for three minutes. Finally, the diphasic supernatant was sucked off, as much as possible, leaving about 1 ml of sediment or suspension for microscopic analysis. Identification of eggs of helminth was done using the shape and size, aided by the diagnosis of intestinal parasites bench aids [17]. A light microscope was used to count the eggs using a magnification of 40. The data was analyzed in Microsoft Excel using descriptive statistics.

## 3. Results

A total of 40 soil samples were used for this study. Two samples (2) were collected from the Teaching and Research Farm of the University of Cape Coast, where these soils are normally used for pot experiments. Twenty-five (25) samples were collected from homes and two (2) samples from hostels. In addition, seven soil samples (7) were collected from schools. Further, two samples (2) were collected from homes that have not been landscaped with transported soil (in other words, the garden or vegetation is growing on the natural soil in situ). Finally, two samples (2) were collected from vehicles transporting black soils to clients.

Roundworm was found in 38 (95%) of the samples. It was absent in one of the samples from the two homes that did not use imported black soil and in one of the samples from schools (Table 1). Hookworm was found in 31 samples (ca. 78%), while whipworm was found in only 10 (25%) of the samples. Roundworm was found to be the dominant helminth, with a total of 98 eggs in all samples, with a mean of approximately 2 and a maximum of 6 eggs g^−1^ soil, followed by hookworm (Table 1). Median values suggest that about 50% of the samples had more than 2 roundworm eggs g^−1^ soil, and more than 1 hookworm egg g^−1^ soil. Whipworm had a median value of zero and a maximum of one egg observed per infected sample. All the helminth egg values showed negative kurtosis and positive skew. The maximum number of helminth eggs (6) was observed in the fresh soil samples obtained from dealer vehicles.

**Figure 1:**
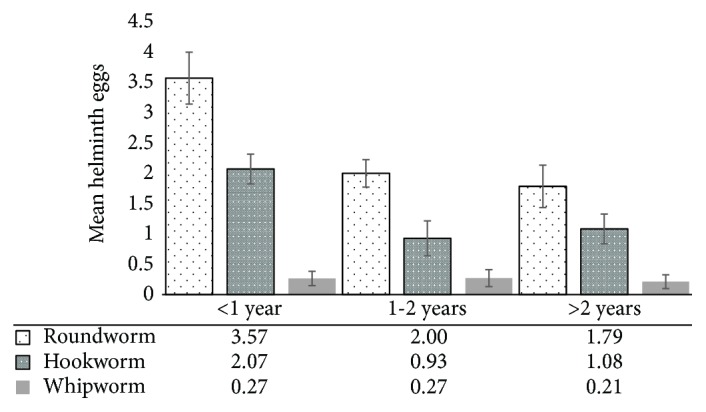
Mean concentrations of helminth eggs (g^−1^ soil) according to duration of use of black soil (error bars are standard errors).

When the analysis was done according to the duration of use of the black soil, the pattern observed for the total samples was repeated. That is, the mean number of eggs for roundworm was the largest (approximately 4 eggs g^−1^ soil), followed by hookworm (Figure 1). There were substantial differences between the mean eggs of roundworm and hookworm within and between the time of use but not for whipworm. Mean eggs for roundworm were the largest for black soil used for less than a year, followed by black soil used between one and two years. For hookworm, the lowest mean eggs were observed for black soils used between one and two years. However, mean whipworm egg count did not differ substantially across the different times of use of black soils.

In addition, with maximum number of eggs counted in samples grouped according to duration of use, roundworm recorded the largest in black soils used under a year and the least in soils used between one and two years (Table 2). The only samples in which roundworm eggs were not observed were in those used for more than two years. Hookworm had a maximum of three eggs g^−1^ soil for all the three durations of use of black soil samples. The median values indicate that larger values of helminth eggs were observed for black soil samples with lower period of use. The largest total number of eggs were 50 (for roundworm, observed for samples used under a year), 29 (for hookworm, observed for samples used under a year), and 4 (for whipworm, observed for samples used under a year).

## 4. Discussion

The results of the current study show that the so-called black soil can be an unsuspected source of STH infection. These soils are currently being used for landscaping (homes, schools, playgrounds, and urban or public spaces), potted plants, home gardening, and plant-based experiments. Sometimes, urban farmers also use these soils in areas with poor or degraded soils. Ghana has made considerable progress in improving environmental sanitation, which, in the long term, is effective for controlling STH. However, open defecation is still widely practiced in both urban and rural areas [10]. The practice is very common in rural settings, urban fringes, and coastal communities. Generally, improvements in environmental sanitation are slow in most low income countries [18]. Soil-transmitted helminthiases are classified among neglected tropical diseases and diseases of poverty [3]. In Ghana, the black soils used in the current study are largely collected from rural areas and urban fringes, which suggest a potential for faecal contamination.

Unlike other pathogens, STH can survive in various environmental media, especially soil, for months to years if conditions are favourable [6]. Moist, shady soil conditions are suitable for embryonation of the eggs, which can occur over a period of five to eighteen days depending on the kind of helminth [1]. Embryonation is prerequisite for the eggs to become infective. Subsequent to embryonation, the eggs can remain infective for up to 10 years or more if environmental conditions are favourable [19]. This suggests that any contaminated soil can remain a potential source of infection for a long time. In the current study, even black soils used for more than two years still contained substantial loads of helminth eggs, though lower than the samples from soils with lower duration of use. The conditions under which black soils are used in Ghana provide alternating favourable and unfavourable conditions for increased persistence and potential for increased infectiveness of helminth eggs in the soils.

In 2015, about 10 million Ghanaian children (both school-aged and preschool-aged) required preventive chemotherapy for soil-transmitted helminthiases based on the Global Health Observatory data [10]. This suggests that STH infection is still a major public health challenge in Ghana. While periodic survey of prevalence and intensity of STH infections is crucial [11, 18], it is equally important to identify potential sources of infections as part of control strategy. The results in the current study show that even children from nonpoor households (with good water and sanitation conditions) can still pick up STH from schools, homes, public places, and other unsuspected sources. Humans can ingest helminth eggs by (i) eating contaminated foods, especially vegetables that are poorly washed, peeled, or cooked, (ii) using contaminated water, and (iii) direct ingestion of contaminated soil (geophagia), which is common for children [1, 4, 5, 11, 20]. In the current study, sources of samples included schools and domestic landscaped surfaces where children and other users can come into direct contact with soil and potentially get infected. Thus, black soils transported for various purposes ought to be considered as part of overall STH infection control strategy.

Roundworm was found to be the dominant STH in the samples studied. Roundworm is the most prevalent helminth in Ghana, accounting for 52% of all helminths' infections [21]. It is also the most common STH infection in tropical countries, coastal communities in West Africa, and the world [16]. Amoah [16] studied helminth loads in wastewater used for irrigation and the soils from fields irrigated with the wastewater in Kumasi (Ghana). He reported that helminth loads in the soil were significantly larger than in the wastewater used for irrigation, suggesting a potential for accumulation. Amongst the helminths' eggs identified in the soils, roundworm was significantly higher than all the other worms. The mean concentrations of helminth eggs reported by Amoah [16] are greater than those found for the samples in the current study. However, the mean concentrations of helminth eggs based on the duration of use of black soil samples in the current study were greater than those reported by Amoah [16]. The observed reduction in helminth egg concentration over time in the black soils in the current study is not immediately clear. However, normally, the quantity of pathogens in human excreta (except bacteria) begins to reduce after excretion due to death, loss of infectivity, or inability to multiply as a result of less favourable environmental conditions outside the human host [20]. The observed reduction in helminth eggs over time might be due to death of helminth eggs through ultraviolet radiation and desiccation as a result of longer exposure to sunlight and dry conditions (especially during dry seasons) under longer durations of use [16, 20]. This can occur due to regular or continuous working of the soil which can bring some of the eggs to the surface and the fact that most landscaped areas are rarely watered during the dry season. It is also probable that eggs can be washed deeper into the soil by rain or irrigation, offsite by erosion (depending on site conditions), and removed by harvesting or weeding plant parts to which eggs might be attached [4, 16, 20]. Lysimeter experiment by Storey and Phillips [22] showed that continuous water application or rain can cause Ascaris eggs to move over a distance of 2.1 cm deeper into the soil in 72 hours and egg persistence increases as they move into deeper layers. Ascaris has the greatest persistence in soil [20], where it can survive between two and seven years or longer under favourable conditions [16, 23]. Hence, it is not surprising that Ascaris was the most abundant in the current study. Thus, in the absence of reintroduction of helminths, the reasons given above can account for the observed reductions in the number of helminth eggs with increasing duration of use of the black soils. Like all other pathogens, helminths are persistent in soils when the conditions are favourable [16, 20]. Overall, the current study shows that use of black soils for landscaping, potted plants, home gardens, and even experiments can pose a risk of STH infection.

## 5. Conclusion

Soil-transmitted helminth (STH) is among the major burdens of neglected tropical diseases. It is a major public health challenge in tropical low income countries. In Ghana, prevalence and infection intensities of STH are large due to poor environmental sanitation practices and conditions. Chemotherapy is the main control measure, while improvement in environmental sanitation (including access to clean water) is recommended as a major route to reducing or eliminating helminth infections. The current study shows that black soils transported and used as growth media for various horticultural purposes can pose potential risk of STH infection. Samples collected from black soils used for various purposes contained substantial loads of helminth eggs. Roundworm was dominant, followed by hookworm and whipworm. The study also shows that the helminth egg concentration reduces with duration of use of the black soils but it is not entirely clear why this is so. It is therefore important to include the analysis and treatment of black soils as part of helminth control strategy. It is also crucial for government to support and encourage the development of a strong growth media market or industry to limit the use of black soils. Finally, sources and use of black soils can be regulated to help reduce the risk of helminth infections.

## Figures and Tables

**Table 1:** Descriptive statistics of concentrations of helminth eggs (g^−1^ soil) in black soil samples (*n* = 40) and number of samples per sample category in which helminth eggs were present.

Statistic	Roundworm	Hookworm	Whipworm
Mean (95% confidence interval)	2.45 (1.9639,2.9361)	1.38 (1.0442,1.7158)	0.25 (0.10928,0.39072)
Median	2	1	0
Standard deviation	1.52	1.05	0.44
Minimum	0	0	0
Maximum	6	3	1
Sum	98	55	10

Presence^*∗*^			

Homes (excluding those without black soil), *n* = 25	25	22	7
Schools (*n* = 7)	6	4	1
Hostels (*n* = 2)	2	1	0
Homes (without black soil), *n* = 2	1	0	0
Dealer vehicle (*n* = 2)	2	2	2
UCC (*n* = 2)	2	2	0
Total	38	31	10

*∗* indicates number of samples in each category found positive for the respective helminth eggs.

**Table 2:** Selected descriptive statistics of helminth eggs (g^−1^ soil) according to age of use of top soil (*n* = 15 for less than one-year soils, 11 for one to two years' soils, and 14 for more than two years' soils).

	Median	Maximum	Minimum	Sum
Ascaris				
<1 yr	3.5	6	1	50
1-2 yrs	2	3	1	26
>2 yrs	1.5	4	0	25
Hookworm				
<1 yr	2	3	1	29
1-2 yrs	1	3	0	13
>2 yrs	1	3	0	13
Whipworm				
<1 yr	0	1	0	4
1-2 yrs	0	1	0	3
>2 yrs	0	1	0	3
